# An Evaluation of Factors Contributing to Primary Nonresponse to Biological Therapy in Patients with Spondyloarthropathy: A Retrospective Cohort Study*

**DOI:** 10.5152/ArchRheumatol.2026.25237

**Published:** 2026-04-10

**Authors:** Emel Oğuz Kökoğlu, Hüseyin Kaplan, Melih Kızıltepe, Tuğba Kahraman Denizhan, Celil Barlas Cengiz, Abdurrahman Soner Şenel

**Affiliations:** 1 Division of Rheumatology, Department of Internal Medicine, Kayseri City Education and Research Hospital, Kayseri, Türkiye; 2 Department of Rheumatology, Aksaray Training and Research Hospital, Aksaray, Türkiye; 3 Division of Rheumatology, Department of Internal Medicine, Erciyes University Faculty of Medicine, Kayseri, Türkiye

**Keywords:** Spondylarthropathies, biological therapy, primary nonresponse, treatment failure

## Abstract

**Background/Aims:**

Primary nonresponse to biological therapy in patients with spondyloarthropathy (SpA) is a considerable challenge. This study sought to identify the factors associated with primary nonresponse to biological therapy in a cohort of patients diagnosed with SpA.

**Materials and Methods:**

A retrospective analysis was performed on 261 patients with SpA who underwent at least 1 biological therapy. The incidence of primary nonresponse to any biological treatment was evaluated, and various clinical and demographic factors were analyzed for their potential associations with treatment outcomes.

**Results:**

The study found that the frequency of primary nonresponse to any biological disease–modifying antirheumatic drugs (bDMARDs) was 15.3% in the study population. In the logistic regression analysis of baseline parameters, presentation with arthritis at diagnosis was identified as a significant predictor of primary nonresponse (odds ratio: 2.113, 95% CI: 1.069-4.175, *P* = .031). Beyond this baseline predictor, primary non-responders significantly differed from responders by having higher frequencies of concomitant fibromyalgia (FMS) treatment (*P* = .047) and higher terminal C-reactive protein (CRP) levels (*P* = .030), as well as variations in acute-phase reactants, presence of psoriasis or enthesitis at diagnosis, and treatment-related factors such as bDMARDs duration.

**Conclusion:**

This study identifies baseline arthritis as a significant predictor of primary nonresponse to bDMARDs in SpA patients. Additionally, the presence of FMS and elevated CRP levels during follow-up are key clinical characteristics associated with the non-responder phenotype. These findings highlight the need for more objective assessment tools to distinguish true biological treatment failure from comorbid pain syndromes and the influence of disease phenotypes.

**Main Points**

- Primary nonresponse to biological disease–modifying antirheumatic drugs (bDMARDs) is a situation encountered at certain rates in patients with SpA, and identifying the factors associated with this may positively influence treatment strategies.
- Patients with and without primary nonresponse differed in terms of acute-phase reactants at various time points, accompanying fibromyalgia (FMS), manifestations of arthritis, psoriasis, and enthesitis at the time of diagnosis, and certain treatment parameters such as the number, duration, and current usage rates of bDMARDs therapy.
- Presentation with arthritis at diagnosis served as a significant predictor of primary nonresponse, whereas concomitant FMS and higher terminal C-reactive protein levels are key clinical features that characterize the non-responder phenotype.
- Recognition of these factors may support individualized treatment decisions and closer monitoring in patients at an increased risk of nonresponse.

## Introduction

Spondyloarthritis (SpA) is a chronic inflammatory rheumatic disorder that predominantly affects the axial skeleton and peripheral joints, leading to considerable pain and disability. The disease may also present with extra-articular manifestations.^1^ The primary goal of managing SpA is to enhance the patient’s health-related quality of life by controlling symptoms and inflammation, preventing structural damage, and preserving function and social participation.^2^ Current recommendations emphasize a combination of non-pharmacological and pharmacological treatments, including non-steroidal anti-inflammatory drugs (NSAIDs), conventional synthetic disease-modifying antirheumatic drugs (csDMARDs), biological disease–modifying antirheumatic drugs (bDMARDs) such as tumor necrosis factor alpha (TNF-α) blockers, targeted synthetic DMARDs (tsDMARDs), and glucocorticoids.^2^ Biological disease–modifying antirheumatic drugs have revolutionized the treatment of SpA in recent years. bDMARDs, specifically TNF-α inhibitors (TNFi), and anti-interleukin (IL)-17A antibodies are effective in patients with SpA, reducing disease activity and mitigating joint-damaging inflammation.^1^^,^^3^ Approximately 20%-30% of patients with SpA discontinue biologic therapy because of either nonresponse or inadequate response to treatment.^^4^^In addition to treatment discontinuation due to safety concerns or intolerance, treatment failure can be categorized into 2 types based on the timing of occurrence: primary nonresponse, which is characterized by a lack of response or efficacy within the first 6 months of treatment initiation, and secondary nonresponse, where an initial response is observed within 6 months but is subsequently lost, indicating a decline in efficacy over time. The complexities underlying primary nonresponse to bDMARDs, particularly TNF antagonists, in patients with SpA represent a significant challenge in rheumatology. Understanding the factors associated with primary nonresponse can help clinicians make informed decisions regarding treatment strategies and potentially improve patient outcomes. While numerous studies^5^^-^^7^ have evaluated predictors of bDMARDs response, early treatment failure, and primary versus secondary nonresponse in SpA cohorts, the present study may offer value by examining a mixed axSpA and pSpA cohort with real-world data.

This study primarily aimed to evaluate the frequency of primary nonresponse to bDMARDs in a SpA cohort and to investigate potential early clinical predictors. Furthermore, the phenotypic characteristics of non-responders were delineated by analyzing the associations between follow-up data and clinical outcomes, aiming to distinguish between initial risk factors and longitudinal disease markers.

## Materials and Methods

### Study Design

This retrospective observational cohort study aimed to evaluate the factors associated with primary nonresponse to bDMARDs. This study was approved by the local ethics committee of Erciyes University (Ethics Committee Decision No.: 2024/279, date: December 4, 2024). Due to the retrospective nature of this study, the Ethics Committee waived the requirement for informed consent.

### Patient Population

The medical records of 371 patients diagnosed with axial SpA (axSpA) or peripheral SpA (pSpA) according to the Assessment of SpondyloArthritis international Society (ASAS) criteria^8^ were reviewed. These patients were regularly monitored with long-term check-ups at the Erciyes University Medical Faculty Internal Medicine Rheumatology Outpatient Clinic between January 2012 and December 2024. The study included 261 patients with a history of bDMARDs use. Patients who had never used bDMARDs, those who had used them for less than 6 months, and those with incomplete data on treatment response parameters were excluded from the study.

### Data Collection

Both manual files and electronic hospital records related to each patient’s visit to the rheumatology outpatient clinic were independently reviewed, starting from the last visit. Various disease-related parameters were systematically evaluated at the time of diagnosis, initiation of bDMARDs therapy, and final assessment.

During the patients’ final visit, data were gathered on the duration of the disease and sociodemographic characteristics (including age, sex, obesity status, educational attainment, marital status, place of residence, and smoking status). Additionally, the presence of comorbidities (such as hypertension, fibromyalgia [FMS], diabetes mellitus, asthma, hyperlipidemia, hypothyroidism, coronary artery disease, chronic obstructive pulmonary disease, chronic kidney disease, and malignancy), additional rheumatologic diseases, and the use of complementary or alternative medicine were recorded. Evidence of comorbidities was gathered from the medical history and treatment information in patient records, as well as medication reports and prescriptions issued using International Classification of Diseases (ICD) diagnosis codes from electronic medical records. The potential impact of concomitant treatments used for comorbidities on primary nonresponse to bDMARDs therapy was also evaluated. However, no reassessment was performed according to any classification criteria to confirm the comorbidities.

At the time of diagnosis, several parameters were considered, including age at diagnosis, age when symptoms first appeared, delay in diagnosis, and initial symptoms exhibited by the patients (such as arthritis, dactylitis, and enthesitis). Additionally, extra-articular manifestations (such as uveitis, psoriasis, and inflammatory bowel disease) and laboratory findings (acute-phase reactants and human leukocyte antigen-B27 [HLA-B27] positivity) were recorded. Rheumatology specialists in this clinic evaluated sacroiliac joint radiographs and magnetic resonance imaging of the sacroiliac joints at the time of diagnosis. Based on this evaluation, the patients were classified as having either axSpA or pSpA.

### Evaluation of Biological Disease–Modifying Antirheumatic Drugs

Both past and current bDMARDs therapies were recorded systematically. The documentation included the initiation date of each bDMARDs, age at onset of bDMARDs treatment, treatment duration for each bDMARDs, cumulative duration of bDMARDs treatment, line of the last bDMARDs used, sequence of bDMARDs with primary nonresponse, current bDMARDs status, number of bDMARDs, therapeutic responses, and reasons for discontinuation or modification of each medication (such as primary or secondary nonresponse, allergic reactions, infections, and pregnancy). Data regarding bDMARDs use were obtained by reviewing patients’ medical history records, electronic file data, medication reports, and prescriptions.

### Evaluation of Disease Activity and Treatment Response

The erythrocyte sedimentation rate (ESR) and C-reactive protein (CRP) values of the patients were documented at 3 key points: diagnosis, start of bDMARDs therapy, and final visit. Furthermore, during the last visit, the disease activity scales Bath Ankylosing Spondylitis Disease Activity Index (BASDAI),^9^ Ankylosing Spondylitis Disease Activity Score (ASDAS),^10^ and global Visual Analog Scale (VAS global)^11^ were recorded. Individuals who did not exhibit a response within 6 months following the commencement of treatment initiation were classified as primary non-responders. A clinical response was defined as a 50% reduction or a 20 mm decrease in the BASDAI within a 6-month period compared to the baseline measurement.^12^ Data collected at 3 different time points were compared. Variables obtained at the baseline visit were evaluated to identify predictors of primary nonresponse, whereas parameters from the last visit were used to identify cross-sectional clinical associations with the nonresponse status, thereby ensuring a clear distinction between predictive factors and follow-up characteristics.

### Statistical Analysis

SPSS for Windows version 23.0 (IBM SPSS Corp.; Armonk, NY, USA) was used for statistical analysis. The Shapiro–Wilk test was used to evaluate the normality of the data distribution. Continuous variables are reported as mean ± SD or median and interquartile range (25%-75%), whereas categorical variables are presented as frequencies and percentages. The independent sample *t*-test was used to compare continuous variables that adhered to a normal distribution between patients with SpA exhibiting primary non-responsiveness to any bDMARDs and other patients. In contrast, the Mann–Whitney *U*-test was applied for continuous variables that did not conform to a normal distribution. Categorical variables were compared using the chi-square test. Factors associated with primary nonresponse were identified using logistic regression analyses (univariate and multiple models). A *P*-value of less than .05 was considered statistically significant.

## Results

This study included 261 patients with a history of bDMARDs use. According to the latest visit data, the mean age of the participants was 46.6 ± 11.6 years, with 55.2% being female, and 15.3% were identified as primary non-responders to bDMARDs. No significant differences were observed in parameters such as age, sex, and body mass index between patients with and without a primary response. Among the cohort, 4 patients had a history of malignancy. One patient had previously undergone surgical intervention for thyroid papillary carcinoma, another for stage 1 squamous cell carcinoma of the lung, a third had basal cell carcinoma, and the fourth had undergone surgery for pituitary adenocarcinoma. All patients commenced treatment with bDMARDs after a minimum of 2 years in remission. Regarding additional rheumatic conditions, 12.3% (32) of the patients were diagnosed with familial Mediterranean fever (FMF), 2.7% (7) with connective tissue disease, 4 patients had Behçet’s disease, 2 patients had Sjögren’s syndrome, 1 patient had both FMF and Behçet’s disease, 1 patient had Takayasu arteritis, and 1 patient had eosinophilic granulomatosis with polyangiitis. No correlation was observed between the presence of these additional rheumatic diseases and response to bDMARDs therapy. The frequency of patients undergoing concurrent FMS therapy was higher in the group with primary nonresponse (25% vs. 11.8%, *P* = .047). Additional demographic and clinical findings of the patients at their last outpatient clinic visit, both with and without primary nonresponse, are presented in Table 1.

Upon evaluating extra-articular manifestations at the time of diagnosis, the rate of primary nonresponse was significantly elevated in the presence of concomitant psoriasis (10% vs. 3.2%, *P* = .048). The primary nonresponse rate was greater in patients with initial presenting signs of arthritis (50% vs. 32%, *P* = .045), whereas it was lower in patients presenting with enthesitis (0% vs. 9.5%, *P* = .026). Of the patients, 199 (76.2%) were diagnosed with axSpA, and 62 (23.8%) were identified as having peripheral SpA. In the axSpA cohort, 79.4% were diagnosed with radiographic axial SpA (r-axSpA). Additional demographic and clinical findings of the patients at the time of diagnosis, with and without primary nonresponse, are presented in Table 2.

In a comparative analysis of individuals with and without primary nonresponse, the median duration of bDMARDs usage was 3 years, with a statistically significantly longer duration observed in the group without primary nonresponse (*P* = .002). Among the patients, 59.8% had a history of using at least 1 bDMARDs, 25.3% had used at least 2, 7.7% had used at least 3, 4.2% had used at least 4, 2.7% had used 5, and 0.4% had used at least 6 bDMARDs previously. The proportion of patients who continued first-line bDMARDs was significantly higher among those who did not exhibit primary nonresponse (*P* < .001). Specifically, 155 patients (70.1%) without primary nonresponse remained on first-line bDMARDs. In contrast, 40% of patients without primary nonresponse transitioned to second-line therapy, 20% to third-line therapy, 25% to fourth-line therapy, and 12.5% to fifth-line bDMARDs. Notably, 1 patient did not initiate alternative bDMARDs treatment following a nonresponse to the initial therapy. Within the cohort experiencing primary nonresponse, 62.5% were nonresponsive to first-line bDMARDs, 30% to second-line bDMARDs, and 7.5% to third- and fourth-line bDMARDs, respectively. At the time of the last evaluation, 243 patients (93%) were actively using bDMARDs. The frequency of active bDMARDs treatment was significantly higher in the group without primary nonresponse (*P* = .010). Detailed information regarding the patients’ bDMARDs use is presented in Table 3.

**Figure 1.**
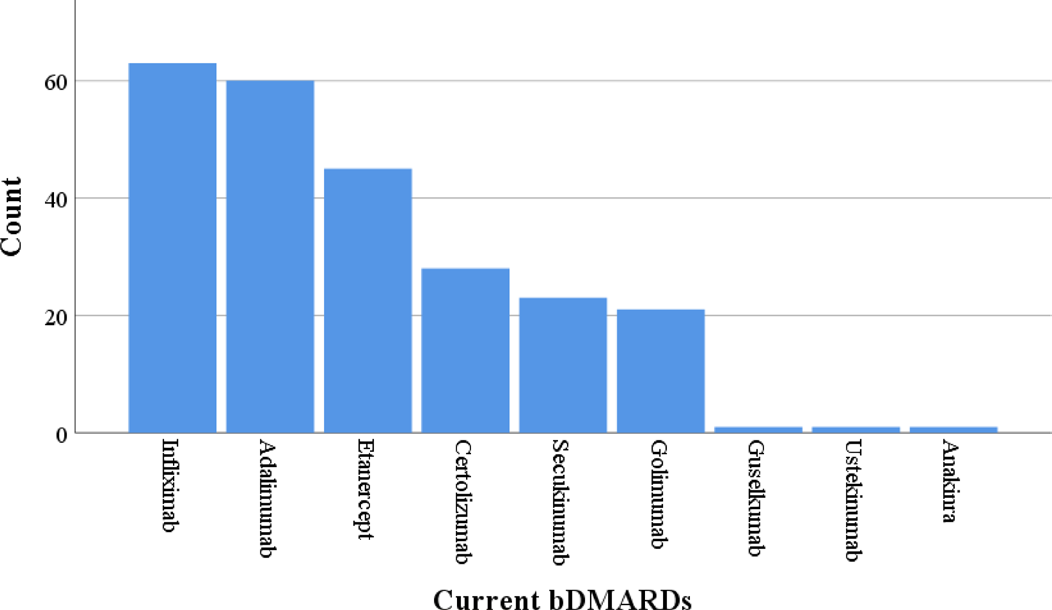
The biological disease–modifying antirheumatic drug that patients were using at their most recent visit.

Data on the last bDMARDs used by the patients is shown in Figure 1.

**Figure 2.**
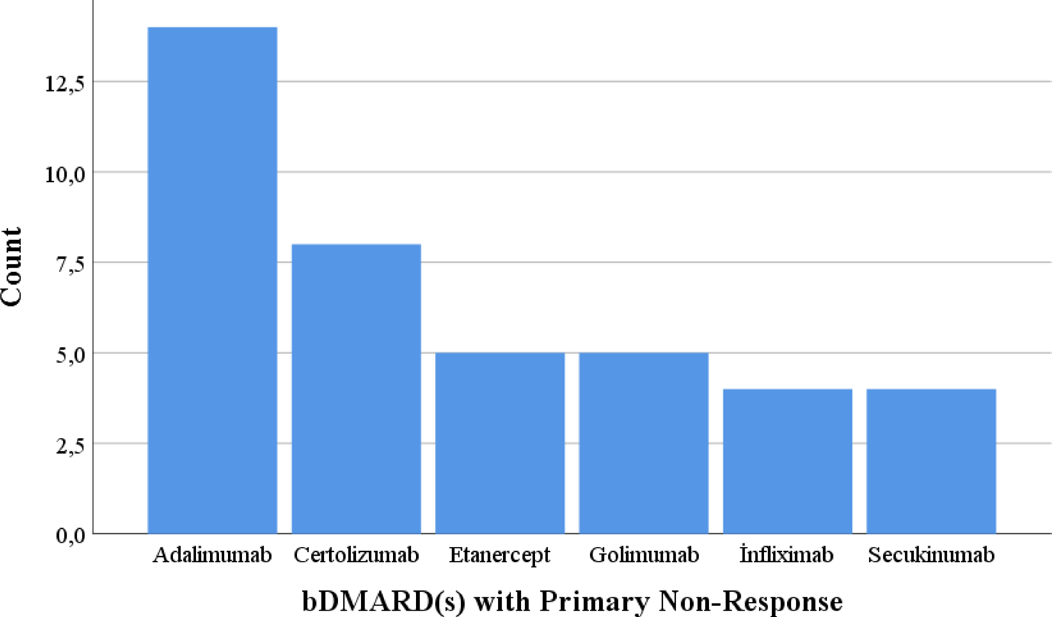
Biological disease–modifying antirheumatic drugs for which patients were primary non-responders.

Among the patients who exhibited primary nonresponse to any bDMARDs treatment, 35% were nonresponsive to adalimumab, 20% to certolizumab, 12.5% to etanercept, 12.5% to golimumab, 10% to infliximab, and 10% to secukinumab (Figure 2).

At the final visit, disease activity parameters were evaluated. The median values for the global VAS were 5 (range: 2-6), for the Ankylosing Spondylitis Disease Activity Score with Erythrocyte Sedimentation Rate (ASDAS-ESR) were 2.3 (range: 1.6-2.9), for ASDAS with C-reactive protein (ASDAS-CRP) were 2.2 (range: 1.3-2.9), and for BASDAI were 3.5 (range: 1.6-5.3) across all patient groups. Global disease activity, as assessed by VAS, was elevated in the primary nonresponse group (median [IQR]: 5 [5-7] vs. 5 [2-6], *P* = .010). Similarly, ASDAS scores were significantly higher among primary non-responders, both for ASDAS-ESR (2.6 [2.1-3.1] vs. 2.2 [1.5-2.9], *P* = .011) and ASDAS-CRP (2.7 [2.1-3.2] vs. 2.1 [1.3-2.9], *P* = .002).

Logistic regression analyses were conducted based on baseline clinical data collected at the time of diagnosis. In the univariate analyses, the following factors were identified as associated predictors of primary non-responsiveness: presentation with arthritis (odds ratio [OR]: 2.113, 95% CI: 1.069-4.175, *P* = .031) and CRP level at the time of diagnosis (OR: 1.016, 95% CI: 1.000-1.031, *P* = .044). Then, candidate predictors were entered into the multiple model. Given that patients with pSpA more frequently present with peripheral arthritis, the SpA subtype was considered appropriate to include in the multiple models to assess its effect. After adjusting for the effects of demographic characteristics (age and sex) and SpA subtypes in the enter model, presentation with arthritis (OR: 2.687, 95% CI: 1.102-6.549, *P* = .030) was identified as a significant predictor of primary nonresponse to bDMARDs (Table 4 ).

## Discussion

In this study, which aimed to identify factors associated with primary nonresponse to biological therapy in patients with SpA who initiated bDMARDs treatment, 15.3% of patients demonstrated a primary nonresponse to bDMARDs. Patients with and without primary nonresponse, acute-phase reactants at various time points, the presence of FMS, arthritis at diagnosis, psoriasis, enthesitis manifestations, and certain treatment parameters (such as the number, duration, and current usage rates of bDMARDs therapy) differed. These findings indicate that the clinical landscape of primary nonresponse is multifaceted. While initial arthritis at diagnosis acts as an early predictor, the persistence of elevated CRP and the higher frequency of FMS treatment at the last visit appear to characterize the long-term clinical profile of these patients, rather than serving as causal predictors. A significant association could not be found between primary nonresponse and factors such as age, sex, obesity status, comorbid diseases (except FMS), use of complementary and alternative medicine, disease duration, age at diagnosis, age of symptom onset, age at the time of bDMARDs initiation, diagnostic delay, dactylitis, IBD, uveitis, ESR, and CRP at diagnosis. To the authors’ knowledge, this study provides significant contributions to the literature by comprehensively evaluating factors associated with nonresponse to biological therapy in SpA patients using a large sample size.

In a longitudinal study involving 147 patients with rheumatoid arthritis (RA), 46 with psoriatic arthritis (PsA), and 37 with ankylosing spondylitis (AS), all with 10-year follow-up data, 35 patients (15.2%) exhibited primary nonresponse to b/tsDMARD therapy. Primary failure was identified as the predominant reason for switching between bDMARDs within the first 12 months.^13^ Additionally, a 54-week open-label prospective study assessed the efficacy and tolerability of transitioning to etanercept 50 mg once-weekly in patients with AS who were resistant or intolerant to infliximab. In this study, primary failure to respond to infliximab treatment was noted in 9% of 23 patients with AS who initiated infliximab therapy.^14^ In this study, 15.3% of patients were identified as primary non-responders to bDMARDs treatment. Adalimumab was the most frequently observed bDMARDs associated with primary nonresponse, accounting for 35% of cases.

In 2004, Rudwaleit et al^^15^^ conducted a pioneering systematic analysis of the parameters associated with clinical responses to anti-TNF therapy in patients with active AS. Their study, which involved 99 patients treated with infliximab or etanercept, found that a favorable response to TNF blockers was significantly correlated with younger age, shorter disease duration, elevated baseline CRP, higher baseline ESR, and lower Bath Ankylosing Spondylitis Functional Index (BASFI) scores.^15^ In a separate investigation involving 220 patients with SpA, the predictors of the highest response rate to anti-TNF therapy were younger age, male sex, elevated ASDAS, higher ESR, and increased CRP.^16^ In a Danish cohort comprising 842 patients with AS who were naïve to TNF-α inhibitors, an investigation was conducted to assess the clinical response, drug survival, and predictors. The study identified male sex, lower baseline VAS scores, and baseline CRP levels exceeding 14 mg/L as factors associated with prolonged drug survival. Additionally, younger age, lower BASFI scores, and elevated baseline CRP levels have been found to be predictors of favorable treatment responses.^^17^^ In a study involving 249 patients with axSpA, approximately 40% of the participants were primary non-responders to TNF inhibitor therapy.^18^ Primary non-responders were identified as being older, more frequently HLA-B27 negative, and exhibiting higher baseline BASDAI scores. In the multiple analysis, older age, HLA-B27 negativity, elevated baseline disease activity, and treatment with soluble TNF receptors were determined to be independent predictors of primary TNFi nonresponse. In this study, no significant differences were observed between patients with and without a primary response in terms of age, sex, and disease duration. Although the ESR value at diagnosis was notably higher in primary non-responders, logistic regression analysis did not reveal a significant relationship. Although baseline CRP appeared to be a significant predictor in univariate analyses, this association was not substantiated in the multiple model. In the present study, patients exhibiting primary nonresponse to bDMARDs therapy demonstrated significantly elevated levels of inflammatory markers and disease activity indices at the final visit, including CRP, ASDAS, VAS, and BASDAI scores. Importantly, these parameters at the last visit should be interpreted as clinical correlates of a non-responsive disease state rather than baseline predictors. Persistent elevation of composite disease activity measures, such as ASDAS, alongside patient-reported outcomes, including VAS and BASDAI scores, signifies a continued disease burden despite exposure to biologic treatments. This observation aligns with previous findings that patients who do not achieve an initial therapeutic response often experience poor long-term disease control and reduced treatment persistence. Notably, the persistent elevation of CRP during follow-up may reflect ongoing objective inflammation, indicating that primary non-responders might represent a more refractory patient subgroup.

Upon evaluation of clinical parameters, it was observed that the frequency of psoriasis at the time of diagnosis was higher. Simultaneously, the frequency of enthesitis was lower in patients who exhibited primary nonresponse. A significant predictor of primary nonresponse was the presentation of arthritis at the time of diagnosis. Although anti-TNF agents are effective treatments for SpA and associated PsA, psoriasis itself has not been consistently identified as an independent predictor of TNFi response in large meta-analyses.^19^ Although psoriasis was more prevalent among patients who did not respond to treatment in this study, it was not identified as a significant predictor in the univariate analyses. In a prospective cohort study assessing the primary clinical predictors of response to anti-TNF therapy, enthesitis was not identified as an independent predictor; however, it was considered within the context of high disease activity.^^2^^0^^In a separate study^^17^^ examining anti-TNF therapy and medication adherence among patients with SpA, it was observed that medication adherence was reduced and treatment modifications were more prevalent in patients presenting with enthesitis. This finding indirectly implies that the presence of enthesitis is correlated with treatment failure. Further research is needed to determine the response/nonresponse to bDMARDs treatment in patients with psoriasis and enthesitis. Numerous observational cohort studies have suggested that the presence of peripheral arthritis in patients with SpA may correlate with varied responses to biological therapy. Registry-based analyses have demonstrated that patients with predominant peripheral arthritis exhibit reduced TNFi drug survival and increased inefficacy rates compared with those with isolated axial disease. This indicates that peripheral manifestations may affect treatment efficacy in real-world contexts.^16^^,^^21^ Furthermore, prospective cohort studies have identified peripheral arthritis as a factor contributing to variability in treatment response. Conversely, Arends et al^16^ identified peripheral arthritis as a significant predictor of the highest response rate to anti-TNF therapy in SpA cohorts. In this retrospective cohort study, the presence of peripheral arthritis at the time of diagnosis emerged as a significant baseline predictor of primary nonresponse to bDMARDs therapy in patients with SpA. Patients presenting with arthritis exhibited significantly higher rates of primary non-responsiveness, a finding that remained robust after adjusting for demographic factors and SpA subtypes. This association is clinically relevant, as peripheral arthritis often reflects a more heterogeneous and multidomain disease phenotype, which may be less amenable to uniform biologic targeting. In contrast to the findings of Arend et al,^16^ the identification of peripheral arthritis as a predictor of a lack of primary response underscores the intricate and heterogeneous nature of the treatment response in patients with SpA. The observed discrepancies in the findings may be ascribed to variations in patient demographics, treatment protocols, or the specific anti-TNF agents employed. These findings support the concept that the initial clinical presentation, particularly peripheral arthritis, may help identify a subgroup of patients with SpA at a higher risk for early biological treatment failure, underscoring the need for closer monitoring and potentially earlier therapeutic optimization or alternative targeted strategies in this population. However, the identification of peripheral arthritis at diagnosis as a predictor of primary nonresponse warrants a cautious interpretation. Given that this cohort includes a heterogeneous mix of patients with both axial and peripheral SpA, this finding may be influenced by phenotype-specific differences in treatment trajectories. In clinical practice, patients with a predominant peripheral phenotype may exhibit distinct response patterns compared to those with purely axial involvement. While multiple models were adjusted for these variables, the risk of phenotype-related confounding remains. It is possible that the presence of arthritis serves as a surrogate marker for a subgroup of patients who are inherently less responsive to traditional bDMARDs pathways or who require different therapeutic targets. Future studies focusing on more homogeneous SpA phenotypes would be essential to validate whether peripheral arthritis remains an independent risk factor across all SpA subtypes.

In a separate investigation, obese patients exhibited a reduced likelihood of responding to anti-TNF-α therapy for axSpA and RA.^22^ A study conducted by Macfarlane et al^^23^^ identified adverse socioeconomic conditions, limited educational attainment, and lack of full-time employment as predictors of a diminished response to initial anti-TNFα treatment. Additionally, comorbidities and poor mental health are correlated with a lack of therapeutic response.^23^ In this study, the frequency of receiving treatment for FMS was significantly higher among individuals exhibiting primary nonresponse than among those who were not. FMS is a prevalent condition characterized by chronic widespread pain and fatigue, and is often diagnosed in individuals with chronic pain.^24^ It can manifest independently or in conjunction with chronic inflammatory diseases. The frequency of FMS in the general population ranges from 2% to 8%, but can exceed 50% in patients with other rheumatic and musculoskeletal diseases.^25^ In patients with axSpA, the reported frequency rates range from approximately 10.8%-38.4%.^26^ In the present study, the frequency of FMS in the entire patient cohort was 13.8%. This frequency was notably higher in patients exhibiting primary nonresponse than in those without such a response (25% vs. 11.8%, respectively). The Assessment of SpondyloArthritis international Society/European Alliance of Associations for Rheumatology (ASAS/EULAR) guidelines recommend evaluating the presence of comorbidities, including FMS, and reassessing the accuracy of the diagnosis in cases of nonresponse to treatment.^^2^^In the literature, axSpA patients are considered difficult-to-treat (D2T) if they experience treatment failure with more than 2 bDMARDs with different mechanisms of action or with more than 3 bDMARDs. Additionally, the presence of signs indicating active or progressive disease, along with the management of symptoms being viewed as problematic by either the rheumatologist or the patient, also contributes to this classification.^27^^,^^28^ In a study conducted by Öğüt et al,^^28^^ which examined the characteristics of 166 patients with D2T axSpA, it was found that the frequency of FMS was elevated, disease activity indices and acute-phase response indicators were increased, and quality of life was diminished in patients with D2T disease. Primary non-responders were more likely to switch between multiple bDMARDs, fitting the definition of difficult-to-treat axSpA, and had a higher frequency of comorbidities, such as FMS, in difficult-to-treat axSpA patients. Given the retrospective nature of this study, patients undergoing concurrent FMS treatment exhibited effects associated with FMS. The potential impact of this situation becomes even more significant if there are patients with FMS who are not receiving treatment. While a significant effect was observed, the retrospective nature of this study precluded a clear distinction between FMS diagnoses made according to the 1990 American College of Rheumatology (ACR) criteria and those made according to the ACR 2010/2016 criteria. A notable consideration in this study is the use of BASDAI improvement as the sole criterion for defining primary nonresponse. While BASDAI is a validated and widely used tool, its reliance on subjective patient-reported symptoms makes it susceptible to influence from non-inflammatory factors, particularly in patients with comorbid FMS. Given the significant association that was observed between FMS treatment and primary nonresponse, the possibility of clinical misclassification cannot be excluded. In some instances, persistent high BASDAI scores may reflect the sensory amplification characteristic of FMS rather than ongoing syndesmophyte progression or active sacroiliitis. Consequently, these findings should be interpreted with caution, as the perceived lack of response might, in a subset of patients, represent the burden of comorbid chronic pain rather than a direct failure of bDMARDs therapy on inflammatory pathways. Fibromyalgia should be evaluated as a parameter in prospective studies focusing on nonresponse to bDMARDs and addressing ASDAS, as this will further clarify its true effect.

In this study, the duration of bDMARDs therapy was notably shorter among patients exhibiting primary nonresponse to the treatment. This finding aligns with real-world registry^29^ and cohort studies^29^^,^^30^ indicating that the lack of initial efficacy is the primary reason for the early discontinuation of biological treatments in SpA. Primary nonresponse, typically identified within the initial months of treatment, inherently leads to reduced drug survival as ineffective therapies are promptly discontinued or switched in accordance with current treatment guidelines. Previous studies^29^^,^^30^ from large registries have suggested that early treatment discontinuation is more frequently related to inefficacy than to adverse events, especially in the early phase of treatment. Therefore, the association between short-term bDMARDs use and primary nonresponse observed in this cohort likely reflects a clinical consequence of early treatment failure rather than a causal relationship. These findings underscore the importance of early treatment response assessment and reinforce the need for timely therapeutic optimization in patients at risk of primary nonresponse.

The strengths of this study include the substantial sample size of 261 patients, which ensured robust statistical power, and the inclusion of comprehensive data encompassing demographic, clinical, and treatment factors. Additionally, the study evaluated both clinical and laboratory parameters as potential predictors and considered comorbidities. However, the limitations of this study include its retrospective design and potentially limited generalizability owing to its single-center nature. Furthermore, there is a lack of a standardized definition of primary nonresponse applicable to all patients, and the study did not account for potential confounding factors, such as medication adherence or lifestyle variables. Owing to the retrospective design of the study, it was not possible to examine the relationship between functional status and radiographic progression, and the absence of genetic or biomarker data also limited the ability to gain further insights into the mechanisms of nonresponse. Moreover, a limitation of the study is the application of the BASDAI score in assessing the response to bDMARDs treatment in patients with both axial and peripheral SpA. The inclusion of both axial and peripheral SpA patients may have introduced clinical heterogeneity. Although these phenotypes were adjusted for in the statistical analysis, the inherent biological and clinical differences between axial and peripheral disease could potentially confound these findings, particularly regarding the role of peripheral arthritis. This diversity within the study population may limit the generalizability of the results to specific SpA subtypes. Finally, the definition of primary nonresponse was based on BASDAI scores. Although ASDAS provides a more objective measure by incorporating acute-phase reactants, it was not the primary metric for the outcome definition. The high prevalence of FMS in this cohort may have led to a potential misclassification of some patients as non-responders due to the subjective nature of the BASDAI tool. Overall, while this study offers valuable insights into the factors associated with primary nonresponse to bDMARDs in SpA, prospective multicenter studies with standardized definitions and more comprehensive data collection are necessary to fully validate these findings and develop predictive models for clinical application.

In conclusion, this study delineates the distinct clinical profile of SpA patients experiencing primary nonresponse to bDMARDs therapy, distinguishing between early predictors and clinical associations observed during follow-up. These findings indicate that peripheral arthritis at disease onset is a significant baseline predictor of primary nonresponse, highlighting the prognostic importance of initial clinical phenotypes. Furthermore, concomitant FMS and persistently elevated CRP levels characterize the non-responder phenotype during the clinical course. However, given the reliance on BASDAI for defining treatment response, the association between FMS and primary nonresponse should be interpreted with caution. The subjective nature of patient-reported outcomes suggests that comorbid pain syndromes may inflate disease activity scores, potentially leading to clinical misclassification. Collectively, these results emphasize that optimizing biologic therapy outcomes in SpA requires a multifaceted approach that integrates baseline characteristics with a careful assessment of comorbidities and inflammatory burden to distinguish true biological failure from the influence of chronic pain.

### Data Availability Statement:

The data that support the findings of this study are available on request from the corresponding author.

### Artificial Intelligence Usage Statement:

No artificial intelligence (AI) or AI-assisted technologies were used in the preparation, writing, or analysis of this manuscript.

### Ethics Committee Approval:

Ethical committee approval was received from the Ethics Committee of University of Erciyes (Approval No.: 2024/279; Date: December 4, 2024).

### Informed Consent:

Written informed consent was obtained from the patients who agreed to take part in the study.

### Peer-review:

Externally peer-reviewed.

### Author Contributions:

Concept – E.OK., H.K.; Design –E.OK., H.K; Supervision – M.K., T.K.D., C.B.C; Resources – E.O.K.; Materials – E.O.K.; Data Collection and/or Processing – E.O.K., H.K., M.K., T.K.D., C.B.C; Analysis and/or Interpretation – E.O.K., H.K; Literature Search – E.O.K., H.K; Writing –E.O.K., H.K.; Critical Review – A.S.Ş.

### Declaration of Interests:

The authors have no conflicts of interest to declare.

### Funding:

The authors declare that this study received no financial support.

## Figures and Tables

**Table 1.** Demographic and Clinical Data of the Patients at the Last Outpatient Clinic Visit

​	All Patients (n = 261)	Patients with Primary Nonresponse (n = 40)	Patients Without Primary Nonresponse (n = 221)	*P*
Age, years	46.6 ± 11.6	49.5 ± 12.9	46.1 ± 11.3	.088
Female, n (%)	144 (55.2)	23 (57.5)	121 (54.8)	.882
BMI, kg/m^2^	28.4 ± 5.2	28.4 ± 4.9	28.8 ± 6.5	.705
Obesity status, n (%)	​	​	​	.278
No	179 (68.6)	24 (60)	155 (70.1)	​
Yes	82 (31.4)	16 (40)	66 (29.9)	​
Disease duration, year	10 (5-13)	10 (7.3-14)	9 (5-13)	.118
Smoking, n (%)	99 (37.9)	17 (42.5)	82 (37.1)	.697
Education, n (%)	​	​	​	.438
Illiterate	5 (1.9)	2 (5)	3 (1.4)	​
Primary school	127 (48.7)	21 (52.5)	106 (48)	​
High school	69 (26.4)	8 (20)	61 (27.6)	​
College/university	60 (23)	9 (22.5)	51 (23.1)	​
Living place, n (%)	​	​	​	.567
Rural	35 (13.4)	7 (17.5)	28 (12.7)	​
Urban	226 (86.6)	33 (82.5)	193 (87.3)	​
Marriage status, n (%)	​	​	​	.222
Single	25 (9.6)	2 (5)	23 (10.4)	​
Divorced	9 (3.4)	3 (7.5)	6 (2.7)	​
Married	227 (87)	35 (87.5)	192 (86.9)	​
Presence of comorbidity, n (%)	137 (52.5)	21 (52.5)	116 (52.5)	1.000
Number of comorbidities	1 (0-2)	1 (0-2)	1 (0-2)	.862
Additional diseases, n (%)	​	​	​	​
Hypertension	56 (21.5)	8 (20)	48 (21.7)	.972
Fibromyalgia	36 (13.8)	10 (25)	26 (11.8)	**.047**
Diabetes mellıtus	35 (13.4)	6 (15)	29 (13.1)	.945
Asthma	25 (9.6)	7 (17.5)	18 (8.1)	.079
Hyperlipidemia	24 (9.2)	4 (10)	20 (9)	.771
Thyroid disorders	20 (7.7)	1 (2.5)	18 (8.6)	.329
CAD	15 (5.7)	1 (2.5)	14 (6.3)	.480
COPD	5 (1.9)	0 (0)	5 (2.3)	.739
CKD	5 (1.9)	1 (2.5)	4 (1.8)	.568
Malignancy	4 (1.5)	1 (2.5)	3 (1.4)	.488
Presence of additional rheumatic comorbidity, n (%)	48 (18.4)	7 (17.5)	41 (18.6)	1.000
Use of complementary and alternative medicine, n (%)	81 (31)	16 (40)	65 (29.4)	.252
ESR, mm /h (0-20)	11 (6-21)	16 (6-23)	10 (6-20.5)	.069
CRP, mg/L (0-5)	3.1 (1.2-7)	4.6 (2-10.1)	2.7 (1.1-6.3)	**.030**

Continuous variables are presented as mean ± SD. Bold emphasis in *P*-values indicate statistical significance.

BMI, body mass index; CAD, coronary artery disease; COPD, chronic obstructive pulmonary disease; CKD, chronic kidney disease; CRP, C-reactive protein; ESR, erythrocyte sedimentation rate; IBD, inflammatory bowel disease.

**Table 2.** Demographic and Clinical Data of the Patients at the Time of Diagnosis

​	All Patients (n = 261)	Patients with Primary Nonresponse (n = 40)	Patients Without Primary Nonresponse (n = 221)	*P*
Age at diagnosis, years	37 (28-45)	37.5 (29-47)	36 (28-45)	.439
Age at symptom onset, years	30 (21-39.5)	30 (20.5-43)	30 (21-38)	.600
Diagnostic delay, years	3 (0-8)	4 (0.3-9.8)	3 (0-7)	.497
Arthritis	91 (34.9)	20 (50)	71 (32.1)	**.045**
Extra-articular manfestations, n (%)	​	​	​	​
IBD	22 (8.4)	3 (7.5)	19 (8.6)	1.000
Psoriasis	11 (4.2)	4 (10)	7 (3.2)	**.048**
Uveitis	27 (10.3)	2 (5)	25 (11.3)	.394
Dactylitis	29 (11.1)	4 (10)	25 (11.3)	1.000
Enthesitis	21 (8)	0 (0)	21 (9.5)	**.026**
HLA-B27 positivity, n (%)	​	​	​	.183
Negative	133 (51)	18 (45)	115 (52)	​
Positive	121 (46.4)	22 (55)	99 (44.8)	​
Unknown	7 (2.7)	0 (0)	7 (3.2)	​
SpA subtypes, n (%)	​	​	​	.841
axSpA	199 (76.2)	30 (75)	169 (76.5)	​
pSpA	62 (23.8)	10 (25)	52 (23.5)	​
ESR, mm /h (0-20)	17.5 (8-37)	31 (11-44)	16 (8-33.5)	**.046**
CRP, mg/L (0-5)	8.6 (3.4-20.5)	14.1 (3.5-38.7)	8.4 (3.4-18.4)	**.049**

Continuous variables are presented as mean ± SD. Bold emphasis in *P*-values indicate statistical significance.

axSpA, axial spondyloarthritis; CRP, C-reactive protein, ESR, erythrocyte sedimentation rate; HLA-B27, human leukocyte antigen-B27; IBD, inflammatory bowel disease; pSpA, peripheral spondyloarthritis; SpA, spondyloarthritis.

**Table 3.** Treatment-Related Data of the Patients at Their Last Outpatient Visit

​	All Patients (n = 261)	Patients with Primary Nonresponse (n = 40)	Patients Without Primary Nonresponse (n = 221)	*P*
Age at the time of bDMARDs initiation, years	40.3 ± 11.8	44.2 ± 11.9	39.3 ± 11.7	.129
bDMARDs treatment duration, years	3 (1.5-6.5)	2 (0.5-4.5)	3 (1.5-7)	**.002**
Line of the last bDMARDs used, n %	​	​	​	**<.001**
1.	156 (59.8)	1 (2.5)^a^	155 (70.1)^b^	​
2.	66 (25.3)	16 (40)^a^	50 (22.6)^b^	​
3.	20 (7.7)	8 (20)^a^	12 (5.4)^b^	​
4.	11 (4.2)	10 (25)^a^	1 (0.5)^b^	​
5.	7 (2.7)	5 (12.5)^a^	1 (0.5)^b^	​
6.	1 (0.4)	0 (0)^a^	1 (0.5)^a^	​
bDMARDs sequence with primary nonresponse, n %	​	​	​	–
1.	–	25 (62.5)	–	​
2.	–	12 (30)	–	​
3.	–	3 (7.5)	–	​
4.	–	3 (7.5)	–	​
Current bDMARDs status, n %	​	​	​	**.010**
Taking medication	243 (93.1)	33 (82.5)^a^	210 (95)^b^	​
No medication	18 (6.9)	7 (17.5)^a^	11 (5)^b^	​
ESR at the time of bDMARDs initiation, mm/h, (0-5)	16 (7-34)	25.5 (12-44)	15 (7-33)	.061
CRP at the time of bDMARDs initiation, mg/L, (0-5)	8.8 (3.7-19.9)	10.9 (3.4-29.6)	8.4 (3.7-18.3)	.240

Continuous variables are presented as mean ± SD or median (Q1-Q3) according to normality tests. Bold emphasis in *P*-values indicate statistical significance.

bDMARDs, biological disease–modifying antirheumatic drugs; CRP, C-reactive protein; ESR, erythrocyte sedimentation rate.

^a,b^Statistically significant differences between categorical groups.

**Table 4.** Identification of Factors Associated with Primary Nonresponse Using Data at the Time of Diagnosis

​	**Univariate Model**				**Multiple Model**			
	OR	95% CI		*P*	OR	95% CI		*P*
Demographics and clinical parameters	​	​	​	​	​	​	​	​
Age at diagnosis (years)	1.013	0.985	1.043	.362	1.021	0.986	1.057	.252
Female gender (ref. male)	1.118	0.566	2.208	.748	1.111	0.452	2.732	.818
Age of symptom onset (years)	1.014	0.986	1.043	.332	​	​	​	​
Diagnostic delay	0.999	0.950	1.051	.972	​	​		​
Arthritis (ref. no)	2.113	1.069	4.175	**.031**	2.687	1.102	6.549	**.030**
Dactylitis (ref. yes)	0.871	0.286	2.653	.808	​	​	​	​
Enthesitis (ref. no)	0.000	0.000	-	.999	​	​	​	​
Extra-articular manifestations (ref. no)	​	​	​	​	​	​	​	​
IBD	0.862	0.243	3.061	.818	​	​	​	​
Psoriasis	3.397	0.946	12.195	.067	​	​	​	​
Uveitis	0.413	0.094	1.816	.242	​	​	​	​
HLA-B27 positivity (ref. negative)	1.420	0.720	2.798	.311	​	​	​	​
SpA subtypes (ref. PSpA)	​	​	​	​	​	​	​	​
axSpA	0.923	0.423	2.014	.841	1.362	0.507	3.658	.540
Disease activity parameters	​	​	​	​	​	​	​	​
ESR	1.016	0.999	1.033	.061	​	​	​	​
CRP	1.016	1.000	1.031	**.044**	1.012	0.996	1.029	.143

Bold emphasis in *P*-values indicate statistical significance.

axSpA, axial spondyloarthritis; CRP, C-reactive protein; ESR, erythrocyte sedimentation rate; HLA-B27, human leukocyte antigen-B27; pSpA, peripheral spondyloarthritis; SpA, spondyloarthritis.
